# Comprehensive Pan-Cancer Analysis Reveals the Role of UHRF1-Mediated DNA Methylation and Immune Infiltration in Renal Cell Carcinoma

**DOI:** 10.1155/2022/3842547

**Published:** 2022-05-23

**Authors:** Xiao Pan, Caiqin Li, Yanqun Cai, Susu Wu

**Affiliations:** Taizhou Municipal Hospital, China

## Abstract

Ubiquitin-like PHD and ring finger domain protein 1 (UHRF1) are members of the multifunctional UHRF family, which can participate in DNA methylation change and histone posttranslational change through particular domains and participate in the event and development of tumors. The purpose of this study was to decide the molecular traits and potential medicine-based importance of UHRF1 that helped settle methylated immune infiltration in generalized cancer by carefully studying the relationship between UHRF1 expression and a variety of tumors and to further check for truth the functional role of UHRF1 in kidney-related cancer. A comprehensive analysis of UHRF1 in 33 cancers was performed based on TCGA database. This research involves analysis of mRNA expression profiles, prognostic value, immune infiltration, immune neoantigens, TMB, microsatellite instability, DNA methylation, and gene set enrichment analysis (GSEA). Both immune infiltration and DNA methylation were used to evaluate the importance and method of UHRF1 in renal cancer. The results showed that tumor tissue had higher expression level of UHRF1 than usual tissue. The high expression level of UHRF1 is related to the survival rate of renal cancer. UHRF1 expression was associated with tumor mutation load and microsatellite instability in different cancer types, and enrichment analysis identified terminology and pathways associated with UHRF1. This study showed that UHRF1 plays an important role in the group of objects and development of 33 tumors. UHRF1 may serve as a biomarker of immune infiltration and poor outlook of cancer.

## 1. Introduction

Ubiquitin-like protein containing PHD and RING finger domains 1, UHRF1, a member of the multifunctional nuclear protein UHRF family, has more than two domains, including the ubiquitin-like domain (UBL), the plant homeodomain (PHD), tandem Tudor domain (TTD), SET and RING-related domain (SET and RING associated (SRA)), and RING domain (RING) [1]. UHRF1 is highly expressed in a variety of tumors and can participate in DNA methylation modification and posttranslational modification of histones through specific structural domains, thus regulating gene expression and participating in the occurrence and development of tumors. Studies have shown that UHRF1 plays an important role in the development of various cancers such as lung adenocarcinoma [2], bile duct cancer [3], kidney cell cancer [4], prostate cancer [5], colon cancer [6], and pancreatic cancer [6]. At the same time, UHRF1 has received considerable attention as a promising biomarker and an important mediator of various human cancers [7]. Unfortunately, the functional role of UHRF1 in renal cancer is still not fully understood. Recently, it has been shown that a generalized carcinoma cohort consisting of RCC related genes is an effective tool for assessing genetic alterations in advanced RCC [8]. However, due to the limited genetic analysis information, large-scale studies and attention to the clinical utility of cancer combinations are needed to further explore the intrinsic relationship between RCC and generalized carcinoma [8]. So it is very important to explore the relationship between UHRF1 expression and different molecular level modification in kidney cancer and various tumors.

Epigenetic modification refers to genetic changes in gene expression, including DNA methylation, histone modification of genomic imprinting, chromosome inactivation, and microRNA (miRNA) regulation [9], and can produce heritable phenotypic changes without altering the DNA sequence [10]. Of these, DNA methylation and histone modification are the most important, and their abnormal changes are always associated with cancer [11]. It is important that UHRF1, as part of DNA methylation and histone modification of key regulatory factor, plays an important role in the occurrence of cancer. The research thinks through targeted maintenance DNA methylation mechanism to reverse the abnormal DNA methylation and a variety of tumor therapy effect, which may become the new treatment strategies of liquid and solid tumors. Dysregulation of the epigenome drives abnormal transcriptional programs that promote cancer occurrence and progression. Although defective gene regulation typically affects oncogenic and tumor suppressor networks, tumor immunogenicity and immune cells involved in antitumor responses may also be affected by epigenome alterations. This may have important implications for the development and application of epigenetic therapies and cancer immunotherapies and their combinations. Here, we review the role of key abnormal epigenetic processes, DNA methylation, and posttranslational modification of histones in tumor immunogenicity, as well as the impact of epigenetic regulation of antitumor immune cell function [12].

In this study, we analyzed the association between UHRF1's ubiquitous-expression methylation analysis of tumor-infiltrating immune cells (TIIC) and associated immune markers by data mining from a variety of databases and visualized its prognostic status in ubiquitous-cancer findings UHRF1 affects the prognosis of cancer patients, probably through its interaction with immune infiltration and methylation modification. UHRF1 is carcinogenic in humans, and the expression of UHRF1 is negatively correlated with the survival time of cancer patients. Taken together, these facts suggest that UHRF1 is not only a marker of immune invasion and poor prognosis but also that its methylation may be a candidate and promising therapeutic target for cancer.

## 2. Materials and Methods

### 2.1. Data Collection and Gene Expression Analysis

TCGA (The Cancer Genome Atlas) contains medicine-based and molecular data from multiple cancer patients with 33 different cancer types [13, 14]. We pull out or taken from something else from TCGA database (https://portal.gdc.cancer.gov/) the transcriptome of 33 kinds of cancer-seq (RNA) data by analyzing UHRF1 differentially expressed between tumor and matched normal tissue information UHRF1 expression in different tumors. GTEx (http://gtexportal.org), a tissue bank and data useful thing established by the National Institutes of Health (NIH) Mutual Fund, has studied 53 healthy human tissues from about 1000 individuals through genetic different version, RNA sequencing, and other molecular phenotypes. For parameter selection, we selected the expression data after Log_2_ (TPM) change for mapping.

### 2.2. Prediction-Related Analysis

Gene Expression Profiling Interactive Analysis (GEPIA) is an online platform for parsing RNA sequencing expression data from TCGA and GTEx projects [15]. We used GEPIA's survival module to evaluate and visualize the association between UHRF1 expression and cancer prognosis.

### 2.3. Analysis of Immune Cell Infiltration in Different Types of Renal Carcinoma

We analyzed the immune cell infiltration of UHRF1 gene in KICH, KIRC, and KIRP using a variety of immunoassay databases, including McCluster, EPIC, QUANTISEQ, TIMER, CIBERSORT, and XCELL database.

### 2.4. Correlation Analysis of UHRF1 Gene Expression with Immunoneoantigen, TMB, and Microsatellite Instability

Neoantigen is a new antigen encoded by mutated genes of tumor cells, which is mainly generated by deletion of mutated genes by gene point mutation and fusion of new abnormal proteins which are different from those expressed in normal cells. Based on the immune activity of tumor neoantigens, neoantigen vaccines can be designed and synthesized according to the mutation of tumor cells, and patients can be immunized to achieve therapeutic effects [16]. Here, we used Scanneo to calculate the number of neoantigens in each tumor sample and analyzed the relationship between the expression of UHRF1 and the number of antigens. The correlation between calculation and UHRF1 expression was realized by R software package GGStatsplot. Spearman correlation analysis was used to describe the correlation between quantitative variables without normal distribution. Spearman's correlation analysis was used to describe the correlation between quantitative variables without normal distribution. *P* < 0.05 was considered statistically significant. As a quantifiable biomarker, tumor mutational burden (TMB) can be used to reflect the number of mutations contained in tumor cells [17]. We calculated TMB microsatellite instability for each tumor sample separately using Spearman rank correlation coefficients. Instability (MSI) refers to the emergence of new microsatellite alleles in tumors due to any changes in microsatellite length caused by insertion or deletion of repeating units compared with normal tissue. At the same time, MSI has the potential to become a key predictor of tumor malignancy, efficacy, and prognosis [18]. Spearman rank correlation coefficient is used to analyze the correlation between UHRF1 expression and MSI, and the radar diagram drawn by R software package Ggradart can be used to intuitively show the correlation difference between several data.

### 2.5. Correlation Analysis of UHRF1 Gene Expression and Immune Marker Sets

We analyzed the expression relationship between more than 40 common immune checkpoint genes and UHRF1, extracted these immune checkpoint genes, calculated the correlation between gene expression and immune checkpoint gene expression, and drew a diagonal heat map using R software package GGplot2. Use the diagonal heat map to show the correlation. The upper triangle is the correlation *P* value (color and significance), and the lower triangle is the correlation coefficient. ∗ in the graph represents the significant correlation *P* < 0.05, ∗∗ indicated significant correlation *P* < 0.01, and ∗∗∗ indicates significant personality *P* < 0.001.

### 2.6. Correlation Analysis of UHRF1 Gene with DNA Repair Gene (MMRs) and Methyltransferase

MMRs are intracellular mismatch repair mechanisms and play a key role in identifying and repairing mismatched nucleotides during gene recombination or damage caused by external physical or chemical damage [19]. We evaluated the association between UHRF1 expression and five MMR genes (MLH1, MSH2, MSH6, PMS2, and EPCAM) using expression profile data from TCGA. The relationship between UHRF1 gene expression and DNA repair gene expression in tumor samples was analyzed. DNA methylation is a form of chemical modification of DNA that can alter epigenetic inheritance and control gene expression without altering the DNA sequence. Here, we analyzed the correlation between UHRF1 expression and the expression of four methyltransferases. R software package GGplot2 was used to draw a diagonal heat map to show the correlation. The upper triangle is the correlation *P* value (color and significance), and the lower triangle is the correlation coefficient graph where ∗ represents significant correlation *P* < 0.05, ∗∗ indicated significant correlation *P* < 0.01, and ∗∗∗ indicates significant personality *P* < 0.001.

### 2.7. GSEA of High and Low Expression Levels of UHRF1 Gene in Tumors

We explored enrichment pathways by comparing the median level of UHRF1 expression with GSEA expression in the high and low expression groups. Through mapping KEGG and HALLMARK pathway in the first five of the most relevant enrichment grading based on NES (net) gene ratio and *P* values proves that the enrichment of significant results of KEGG pathway |NES| > 1NOM*P* < 0.05 and FDR *q* < 0.25 of the genome was considered to be significantly enriched [20].

### 2.8. Immunohistochemistry (IHC) Staining Analysis

In order to evaluate the difference in UHRF1 expression at the protein level, we downloaded data from TCGA, GTEx, and HPA (Human Protein Atlas) (http://www.proteinatlas.org/) and analyzed. Among them are UHRF1 protein expression level data and IHC images in normal kidney tissues and three types of kidney cancer tissues, including KICH, KIRC, and KIRP.

## 3. Results

### 3.1. The Expression Level of UHRF1 Is Different in Different Tumors

**Figure 1:**
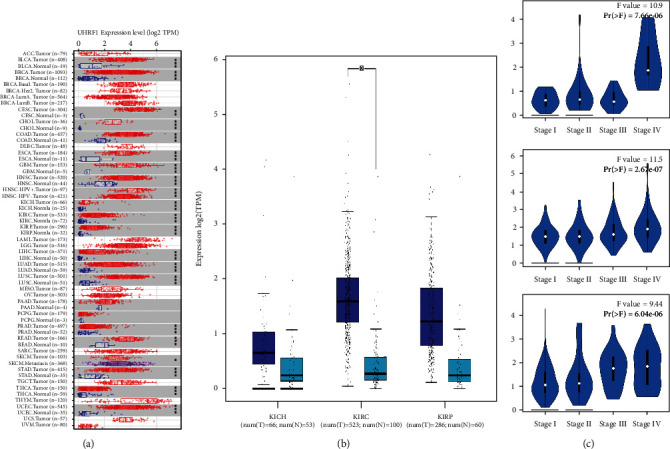
(a) UHRF1 gene expression in different tumors. (b) UHRF1 gene expression in KICH, KIRC, and KIRP. (c) UHRF1 gene expression in different stages of KICH, KIRC, and KIRP.

TIMER2 method was used to analyze the expression of UHRF1 in different types of TCGA. As shown in Figure 1(a), UHRF1 was found in BLCA (urothelial carcinoma of the bladder), BRCA (invasive breast carcinoma), CHOL (bile duct cancer), COAD (colonic adenocarcinoma), ESCA (esophageal cancer), GBM (polymorphogenetic glioma), HNSC (head and neck squamous cell carcinoma), KICH (renal chromogenic cell carcinoma), KIRC (renal clear cell carcinoma), KIRP (renal papillary cell carcinoma), LIHC (hepatocellular carcinoma), LUAD (lung adenocarcinoma), LUSC (lung adenocarcinoma), PRAD (prostate cancer), PEAD (rectal adenocarcinoma), STAD (gastric cancer), THCA (thyroid cancer), UCEC (endometrial cancer) (*P* < 0.001), and CESC (cervical squamous and adenocarcinoma) (*P* < 0.01) which was higher than that of the corresponding control tissue. Meanwhile, we analyzed UHRF1 mRNA expression in KICH, KIRC, KIRP, and normal renal tissue RNA sequencing data by GEPIA2. The results showed that the expression level of UHRF1 mRNA in KIRC was higher than that in nontumor renal tissues (*P* < 0.05, as shown in Figure 1(b)). In addition, the correlation between UHRF1 expression and pathological staging of cancer was observed using the histopathological staging module of HEPIA2, including KICH, KIRC, and KIRP (Figure 1(c), *P* < 0.05), and not associated with other cancers.

### 3.2. Prognostic Correlation Analysis of UHRF1 in Different Stages of Renal Cancer

**Figure 2:**
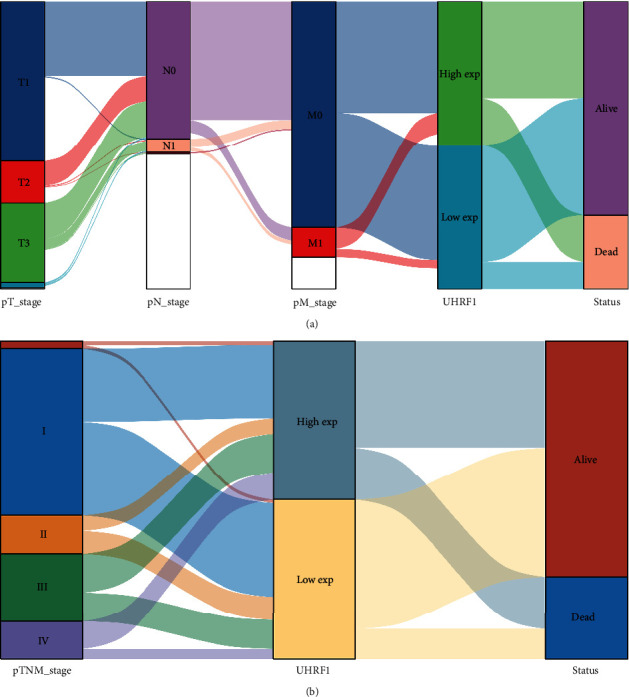
Prognostic relationship between UHRF1 gene and different stages of renal carcinoma.

We analyzed the correlation between the expression level of UHRF1 gene and the prognosis of patients with different stages of renal cancer by drawing a Sankey map and carried out data visualization. The results showed that the correlation between TNM stage and prognosis indicated that tumors showed high expression of UHRF1 gene in the M1 stage. Conclusively, terminal mortality was associated with high expression of the UHRF1 gene, as shown in Figure 2(a). Similarly, UHRF1 gene was highly expressed in stages II, III, and IV of tumors, while UHRF1 gene was expressed low in stage I, and the high expression of UHRF1 gene significantly correlated with the death of patients with renal cancer, while the low expression significantly correlated with the survival of patients with renal cancer, as shown in Figure 2(b).

### 3.3. Construction and Analysis of Prognostic Model between UHRF1 and Clinical Characteristics of Renal Cancer Patients

**Figure 3:**
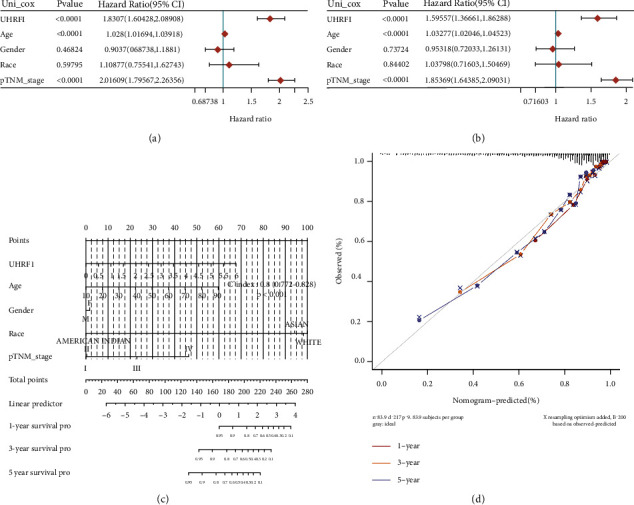
(a, b) Univariate and multivariate Cox analysis of gene expression and clinical characteristics of *P* value, risk coefficient HR, and confidence interval. (c) Nomogram for predicting 1-year, 3-year, and 5-year overall survival in kidney cancer patients. (d) Calibrated graphs of the population survival probability rolograph model, with diagonal dotted lines representing ideal rolographs and red, orange, and blue lines representing observed 1-year, 3-year, and 5-year rolographs.

We obtained RNAseq data and corresponding clinical information of 883 renal cancer patients (KICH, KIRC, and KIRP) from The Cancer Genome Atlas (TCGA) database. First, we performed univariate and multivariate Cox regression analysis and used forest plots through the “forestplot” package to display each variable (*P* value, HR, and 95% CI), as shown in Figures 3(a) and 3(b). We then used the “rms” package to construct nomograms to predict 1-, 3-, and 5-year overall recurrence rates based on the results of a multivariate Cox proportional hazards analysis, and the visualization provided graphical results for these factors, which can be compared with each. Points associated with risk factors were used to calculate the prognostic risk of an individual patient. The *c*-index was 0.8 (0.772-0.828), *P* < 0.001, indicating that the prediction performance of the model was good. Meanwhile, the correction curve showed that the prediction accuracy of the model verified the survival probability of patients with renal cancer in 1, 3, and 5 years was excellent, as shown in Figures 3(c) and 3(d).

### 3.4. Survival Analysis of UHRF1 in Different Tumors

**Figure 4:**
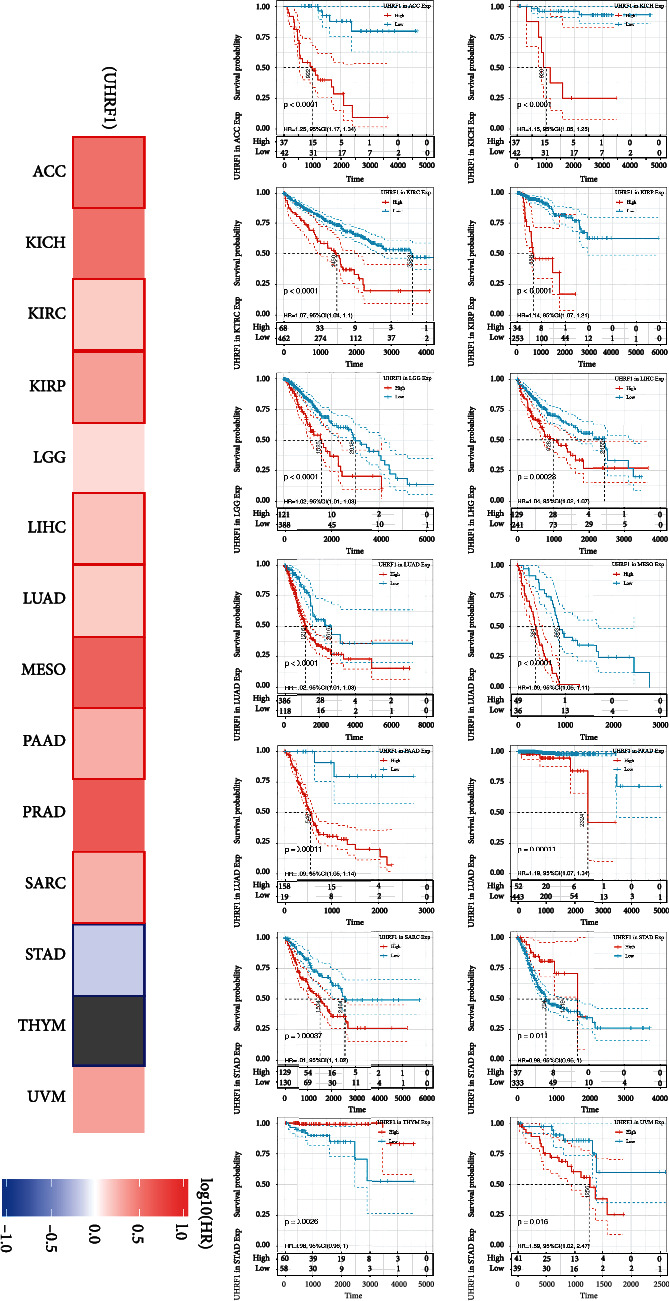
The relationship between UHRF1 gene expression and survival prognosis of various TCGA cancers. We used GEPIA2 tool to compare and analyze the UHRF1 gene high expression group and low expression group of different TCGA tumors and presented the survival map and Kaplan-Meier curve.

We divided tumor cases into high expression group and low expression group by the median expression level of UHRF1 in tumor tissue samples and used tumor data in TCGA to study the correlation between UHRF1 expression and prognosis of patients with different tumors. As shown in Figure 4, in TCGA tumor data, high expression of UHRF1 mRNA was associated with ACC (*P* < 0.0001), KICH (*P* < 0.0001), KIRC (*P* < 0.0001), KIRP (*P* < 0.0001), LGG (*P* < 0.0001), LIHC (*P* = 0.00023), LUAD (*P* < 0.0001), MESO (*P* < 0.0001), PAAD (*P* = 0.00011), PRAD (*P* = 0.00011), SARC (*P* = 0.00037), STAD (*P* = 0.00011), and UVM (*P* = 0.016). The UHRF1 mRNA expression group had a lower survival rate than the low expression group. In contrast, high expression of UHRF1 mRNA in THYM (*P* = 0.0026) was associated with higher survival.

### 3.5. Analysis of UHRF1 Gene and Immune Cell Infiltration

**Figure 5:**
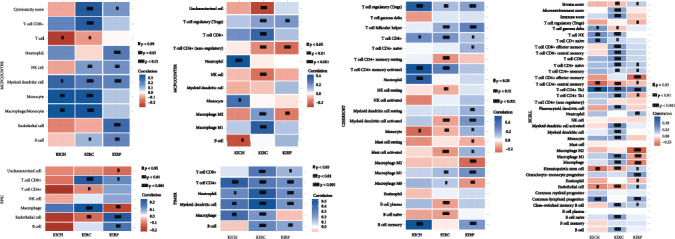
UHRF1 gene distribution in immune-related cells in McCluster, EPIC, QUANTISEQ, TIMER, CIBERSORT, and XCELL databases.

Based on a variety of immunoassay databases, including MCPcounter, EPIC, QUANTISEQ, TIMER, CIBERSORT, and XCELL, we performed immunocell infiltration analysis of UHRF1 gene in KICH, KIRC, and KIRP, as shown in Figure 5. KICH was found in monocyte macrophage/monocyte, none, neutrophil, T cell CD4+, T cell regulatory (Tregs), T cell CD4+ memory activated B cell memory, and T cell CD4+ Th2 which are highly infiltrated. KIRP is located in neutrophil endothelial cell in the above database, endothelial cell, T cell regulatory (Tregs), T cell CD4+ neutrophil myeloid dendritic cell, T cell helper myeloid cell activated macrophage M1 B cell memory, and T cell CD4+ Th2 high infiltration of granulocyte-monocyte progenitor.

### 3.6. Correlation Analysis of UHRF1 Gene Expression with TMB, Immunoneoantigen, and Microsatellite Instability

**Figure 6:**
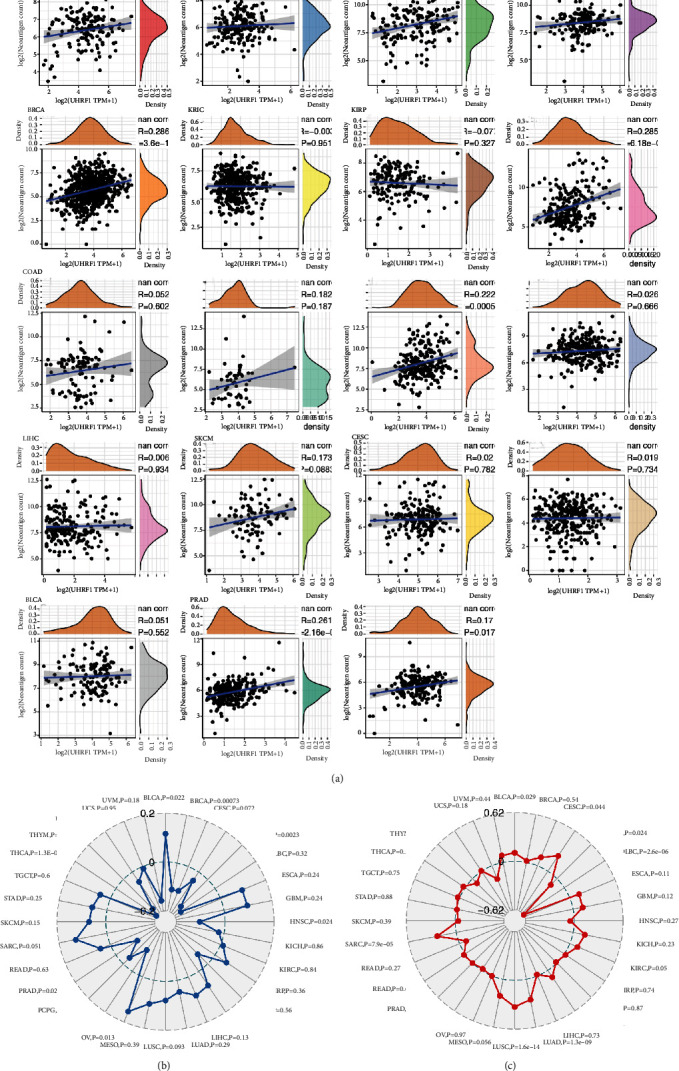
(a) Correlation analysis between UHRF1 gene expression and the number of tumor immune neoantigens. (b) Correlation between UHRF1 gene expression and TMB. (c) Correlation between UHRF1 gene expression and MIS.

In order to determine the relationship between UHRF1 gene expression and tumor immune neoantigens, we counted the number of neoantigens in each tumor sample. By analyzing the relationship between UHRF1 gene expression and the number of neoantigens, as shown in Figure 6(a), UHRF1 gene is closely related to LUAD, BRCA, UCEC, STAD, PRAD, and LGG. At the same time, we analyzed the correlation between UHRF1 gene expression and TMB MIS in various tumors, using Spearman rank correlation coefficient. Studies showed that UHRF1 was positively correlated with TMB in BLCA and OV and negatively correlated with TMB in BRCA, COAD, HNSC, PRAD, and THCA, as shown in Figure 6(b). UHRF1 was positively correlated with TMB in BLCA, LGG, LUAD, LUSC, and SARC. MSI was positively correlated and negatively correlated with MSI in COAD and DLBC (Figure 6(c)). The association between UHRF1 expression and TMB MIS differed significantly between cancer types.

### 3.7. Correlation Analysis of UHRF1 Gene Expression and Immune Marker Sets

**Figure 7:**
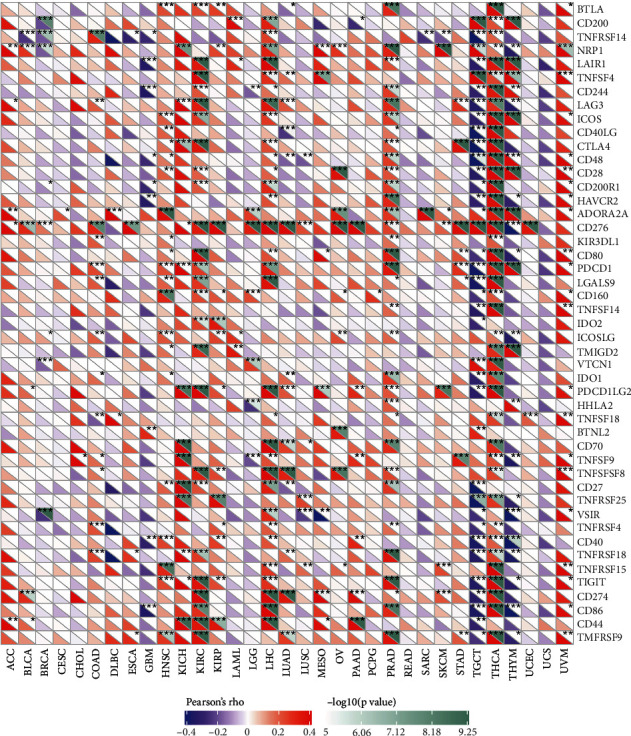
Association between UHRF1 expression and immune marker set: immune marker set in cancer.

The importance of immunosurveillance in determining prognosis of various types of cancer is widely accepted. Moreover, tumors can evade immune responses by using immune checkpoint genes. To determine the association between UHRF1 and the degree of immune invasion in different tumors, we analyzed the association between UHRF1 and immune checkpoint gene expression. In KIRC, UHRF1 expression is similar to BTLA, LAIR1, TNFSF4, LAG3, ICOS, CTLA4, CD276, CD80, PDCD1, LGALS9, TMIGD2, PDCD1LG2, TNFRSF8, TIGIT, CD274, and CD86. There was a positive correlation between the expression of CD44 and TNFRSF9, as shown in Figure 7. These results suggest that UHRF1 overexpression may play an important role in mediating immune evasion.

### 3.8. The Expression of UHRF1 Gene Was Correlated with the Expression of DNA Repair Gene (MMRs) and Methyltransferase in Tumor Samples

**Figure 8:**
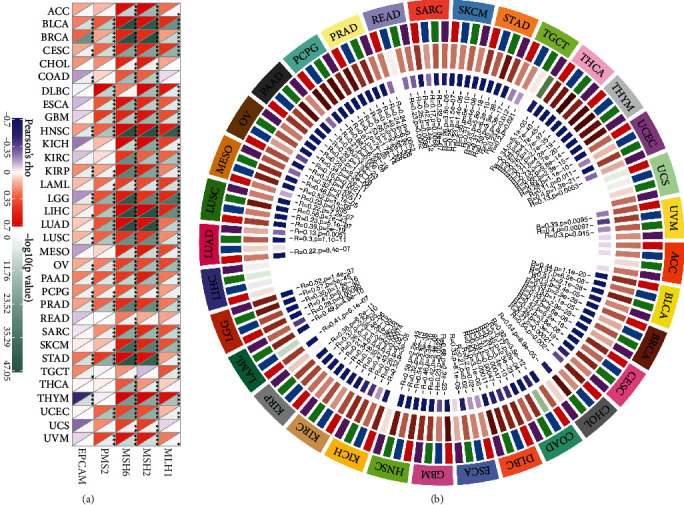
(a) The relationship between UHRF1 gene expression and MMR expression in tumors. (b) The relationship between UHRF1 gene expression and four methyltransferase expression in tumors.

We analyzed the correlation of mismatch repair mechanism gene mutations in 33 tumors in TCGA database. The results showed that UHRF1 gene expression was associated with MSH2 MSH6 gene mutation in a variety of tumors. Among them, KICH KIRC is related to MLH1, MSH2, and MSH6, and KIRP is related to MLH1, MSH2, MSH6, PMS2, and EPCAM mutation as shown in Figure 8(a). At the same time, we analyzed and visualized the correlation between gene expression and expression of four methyltransferases (DNMT1: red; DNMT2: blue; DNMT3A: green; DNMT3B: purple). The results showed that a variety of tumors were correlated with four kinds of methyltransferases, among which KICH KIRC KIRP was positively correlated with methyltransferases. Interestingly, LIHC UCS was not correlated with four kinds of methyltransferases, as shown in Figure 8(b).

### 3.9. GSEA of High and Low Expression Levels of UHRF1 Gene in Tumors

**Figure 9:**
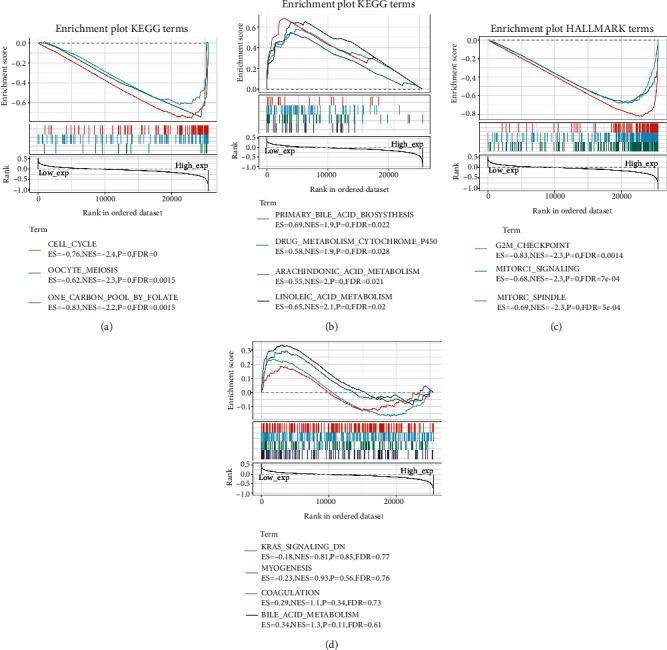
GSEA of UHRF1 in high and low expression samples. Each line represents a specific genome with independent color, with UHRF1 upregulated genes located on the left near the origin of the coordinates and downregulated genes located on the right of the *x*-axis. (a) Gene set enriched by UHRF1 high expression samples in KEGG collection. (b) Gene set enriched in KEGG for UHRF1 low expression samples. (c) Gene set enriched by UHRF1 high expression samples in HALLMARK. (d) Gene set enriched in HALLMARK of UHRF1 low expression samples.

In order to observe the effect of UHRF1 gene expression on tumor, we divided the samples into high and low groups according to gene expression. GSEA was used to analyze the enrichment of KEGG and HALLMARK pathways in the high and low expression groups. KEGG enrichment term indicated that the high expression of UHRF1 was mainly related to cell cycle, including the carbon pool formed by folic acid in oocyte meiosis. The low expression of UHRF1 is mainly related to the biosynthesis of primary bile acids, including drug metabolism cytochrome P450 arachidonic acid metabolism and linoleic acid metabolism, as shown in Figures 9(a) and 9(b). However, HALLMARK is remarkably rich and suggests that the high expression of UHRF1 is mainly related to the G2M checkpoint, including the MTORC1 signal mitotic spindle. The screening criteria are NOM *P* < 0.05 and a genome of FDR *q* < 0.0.06 is considered significant (Figures 9(c) and 9(d)).

### 3.10. Immunohistochemical Analysis of UHRF1 Gene in Renal Cell Carcinoma

**Figure 10:**
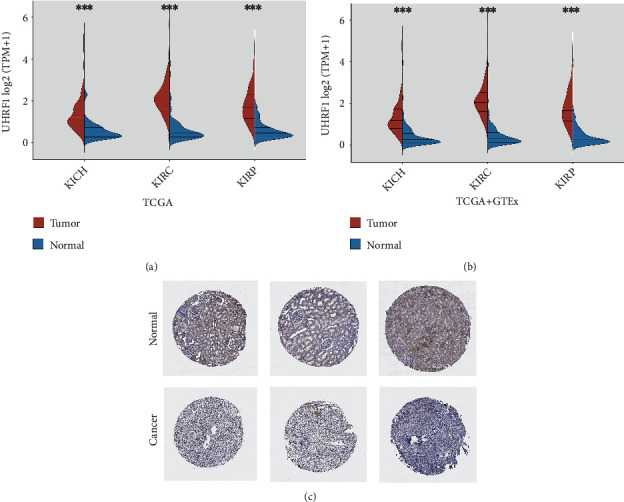
Sample data obtained from TCGA and GTEx and immunohistochemical images obtained from HPA database. (a, b) Comparison of UHRF1 gene expression between normal and tumor tissues in TCGA and GTEx databases. UHRF1 protein expression is significantly higher in KICH, KIRC, and KIRP. (c) Immunohistochemical images of normal kidney tissue and kidney cancer tissue obtained from the HPA database.

In order to explore the immunohistochemical differences between the UHRF1 gene in the three kidney cancer tissues (KICH, KIRC, and KIRP) and normal kidney tissues, we analyzed the IHC results provided by the HPA database. At the same time, we compared the results with UHRF1 gene expression data from TCGA and GTEx databases. The data analysis results of these three databases are consistent with each other. The expression of UHRF1 in normal kidney tissue and the three kinds of kidney cancer tissues in the TCGA and GTEx databases is significantly different (Figures 10(a) and 10(b)). On the other hand, the UHRF1 gene was negative or moderately stained by IHC in normal kidney tissues and the staining morphology was regular, while in the three types of kidney cancer tissues, it was stained moderately or strongly with disordered tissue morphology (Figure 10(c)).

## 4. Discussion

More and more studies have shown that UHRF1 plays a crucial role in the development of cancer, but the comprehensive analysis of UHRF1 in different cancers is still insufficient. Therefore, it is necessary to investigate the expression survival prognosis of cancers with abnormal expression of UHRF1 immune infiltrates in DNA methylation and functional pathways. The aim of our study was to explore the characterization of UHRF1 in generalized cancer and its potential function in renal cancer (KIRP, KIRC, and KICH). As a multifunctional nuclear protein, the biological function of UHRF1 has been proved to be involved in DNA methylation and play an important role in various tumorigenesis [21, 22]. A large number of studies have shown that the differential expression of UHRF1 is highly expressed in a series of human tumors such as breast cancer, cervical squamous cell carcinoma, esophageal squamous cell carcinoma, hepatocellular carcinoma, and colon cancer [23–27]. Meanwhile, UHRF1 is considered to be an important regulator of pancreatic cancer cell proliferation, metabolism, and metastasis [6]. The uHRF1-mediated PI3K/Akt signaling pathway downregulates the bcl-2/Bax expression ratio and promotes caspase-9 expression, which can inhibit the proliferation of retinoblastoma cells and promote apoptosis [28]. The high expression of UHRF1 inhibits a variety of tumor suppressor genes, such as BRCA1, KISS1, and MEG [29–32].

According to the survival prognosis analysis, UHRF1 is mainly associated with the adverse survival of ACC, KICH, KIRC, KIRP, LGG, LIHC, LUAD, MESO, PAAD, PRAD, SARC, STAD, and UVM. In the prognosis of different stages of renal cancer, the high expression of UHRF1 gene was significantly correlated with the death of patients with renal cancer, while the low expression was significantly correlated with the survival of patients with renal cancer. Studies have shown that the natural anticancer drug, epigallocatechin-3-gallate (EGCG), induces a significant decrease in the expression of UHRF1 and DNMT1 in Jurkat cells, upregulated with P16 INK4A, cell cycle G1/S stagnation, and apoptosis [33]. Wotschofsky et al. found that UHRF1 was downregulated by Mir-146A-5p through knockdown and overexpression experiments of miRNA in renal cancer cell lines. The new target gene UHRF1 of dysregulated miRNA is associated with distant metastasis of primary RCC [34]. Alhosin et al. believe that the signaling pathway regulated by UHRF1 in cancer cells will enable us to find new therapeutic targets to inhibit the expression of UHRF1, thus enabling cancer cells to reexpress tumor suppressor genes leading to tumor cell apoptosis [35].

In recent years, immunotherapy has shown higher efficacy in treating tumors. Notably, this study suggests that UHRF1 levels are associated with cancer immunity. Among them, CIBERSORT, an online immune cell analysis tool, has been used in both tumor and nontumor diseases, such as triple-negative breast cancer [36], tendinopathy [37], and myocarditis [38]. In the results of this study, UHRF1 levels are associated with the degree of immune invasion of renal cancer type. Based on the infiltration analysis of six immune cells, we found that UHRF1 level was significantly correlated with the infiltration degree of T cell CD4^+^, Th2 monocyte, macrophage M1, and neutrophil. The Hansen team identified 52 new epitope-specific CD8^+^ from T cell responses in tumor-infiltrating lymphocytes from six RCC patients using a novel high-throughput technique using pMHC polymers. At the same time, they detected that all the new epitopes were restricted by MHCI class [39]. More importantly, these immunogenicity characteristics are critical for the use of neoantigens as immunotherapy-related therapeutic targets and biomarkers for RCC. Liu et al. demonstrated through experiments that UHRF1 downregulation and reduction of DNA methylation and H3K27me3 levels resulted in increased BCL6 expression and promoted Tfh cell differentiation in vitro and in vivo [40]. It is well known that tumor immunotherapy can restore the body's normal antitumor immune response, including monoclonal antibody immune checkpoint inhibitors cancer vaccine therapeutic antibodies and cell therapy. We calculated the correlation with the expression of our target gene by collecting more than 40 common immune checkpoint genes and analyzing their expression relationships with our gene expression. Previous studies have suggested that regulation of tumor immune escape via the RP11-424C20.2/UHRF1 axis plays a different role in the progression of hepatocellular carcinoma (LIHC) and thymoma (THYM) and is associated with IFN-*γ*-mediated CLTA-4 and PD-L1 pathways [41]. Our results showed that UHRF1 upregulation was positively correlated with BLCA, LGG, LUAD, LUSC, SARC, and MSI. MSI is associated with a higher risk of cancer and has different clinicopathological features, including increased TMB and tumor-infiltrating lymphocyte counts [42]. On the other hand, we obtained that UHRF1 was positively correlated with TMB in BLCA and OV, and the combination of tumor mutation load (TMB) and copy number change (CNA) could be used to group a variety of metastatic tumors, and the optimal treatment subgroup could be selected according to the prognosis of different groups [43]. Studies have shown that TMB is a useful biomarker for immune checkpoint blocking (ICB) treatment options in some cancer types [44]. Therefore, our study elucidates the potential role of UHRF1 in tumor immunity and its use as a prognostic biomarker for cancer.

A large number of studies have shown that abnormal epigenetic regulation of gene function is closely related to the occurrence of cancer [45]. Epigenetic mutations (EPimutations) are involved in the earliest stages of tumor formation and are increasingly considered as markers of cancer. Therefore, it is important to explore the genetic changes and methylation of UHRF1. Our results showed that UHRF1 gene expression was correlated with four methyltransferases (DNMT1, DNMT2, DNMT3A, and DNMT3B) in a variety of tumors, and KICH KIRC KIRP was positively correlated with methyltransferase, suggesting that UHRF1 may be an epigenetic driver of renal cancer type. Our results showed that UHRF1 inhibited TXNIP expression by enslating HDAC1 to the TXNIP promoter and mediating deacetylation of histone H3K9, thus confirming that UHRF1 may promote tumor progression through epigenetic regulation of TXNIP in renal cancer [4].

GSEA enrichment analysis showed that the high expression of UHRF1 was mainly related to the cell cycle. Recent studies suggest that UHRF1 knockdown can affect the lung adenocarcinoma (ADC) cell cycle and induce apoptosis, and the results show that UHRF1 upregulation can promote the survival of ADC cells by triggering the cell cycle pathway [2]. At the same time, HALLMARK significantly enriched that the high expression of UHRF1 is mainly related to the G2M checkpoint, including the MTORC1 signaling mitotic spindle. Experiments showed that cancer cells depleted of UHRF1 would activate the DNA damage response pathway, resulting in cell cycle stagnation in G2M and apoptosis dependent on the caspase-8 pathway [46]. Interestingly, previous studies have also suggested that reduced UHRF1 results in cell cycle arrest in G1 and G2 phases [47]. During the S phase of the cell cycle, UHRF1 recognizes CpG sites for hemimethylation through its SRA domain and directs DNA methyltransferase 1 (DNMT1) to these sites to mediate DNA methylation [48–50].

Unfortunately, even though our study integrated a large sample of information from different databases, there are still some limitations. Multiple bioinformatics analyses have provided us with some meaningful insights into the role of UHRF1 in generalized cancer, but biological experiments in vitro or in vivo are needed to validate our results and further mechanistic studies are needed to elucidate the role of UHRF1 expression levels at the molecular and cellular levels. More importantly, we need to explore the effects of UHRF1 on various tumors through mediating tumor immunity and DNA methylation.

In conclusion, this study elucidates the close correlation and prognostic significance of UHRF1 expression in various human cancer pathogenesis. At the same time, UHRF1 has more significant tumor immunity and DNA methylation effects in kidney cancer types. We speculate that UHRF1 may be a novel target for cancer therapy, as it is upregulated in a variety of cancers and is associated with poorer prognosis. In addition, our results provide a potential mechanism by which UHRF1 expression may regulate tumor immunity, DNA repair, and methylation in cancer. Future studies on UHRF1 expression and tumor immune microenvironment and methylation may provide new strategies for tumor immunotherapy.

## 5. Conclusion

In conclusion, this study demonstrates that UHRF1 plays a key role in renal cancer through DNA methylation and the immune microenvironment. By further understanding its functional scope, we can make UHRF1 an effective biomarker for the diagnosis and treatment of renal cancer.

## Data Availability

Publicly available datasets were analyzed in this study. This data can be found here: The Cancer Genome Atlas (https://portal.gdc.cancer.gov/).
